# A High Full Well Capacity CMOS Image Sensor for Space Applications

**DOI:** 10.3390/s19071505

**Published:** 2019-03-28

**Authors:** Woo-Tae Kim, Cheonwi Park, Hyunkeun Lee, Ilseop Lee, Byung-Geun Lee

**Affiliations:** 1 School of Electrical Engineering and Computer Science, Gwangju Institute of Science and Technology, Gwangju 61005, Korea; wtkim@gist.ac.kr (W.-T.K.); cwpark@gist.ac.kr (C.P.); hklee4@gist.ac.kr (H.L.); 2 Korea Aerospace Research Institute, Daejeon 34133, Korea; islee@kari.re.kr

**Keywords:** CMOS image sensors, wide dynamic range, multiple charge transfer, space applications, radiation damage effects

## Abstract

This paper presents a high full well capacity (FWC) CMOS image sensor (CIS) for space applications. The proposed pixel design effectively increases the FWC without inducing overflow of photo-generated charge in a limited pixel area. An MOS capacitor is integrated in a pixel and accumulated charges in a photodiode are transferred to the in-pixel capacitor multiple times depending on the maximum incident light intensity. In addition, the modulation transfer function (MTF) and radiation damage effect on the pixel, which are especially important for space applications, are studied and analyzed through fabrication of the CIS. The CIS was fabricated using a 0.11 μm 1-poly 4-metal CIS process to demonstrate the proposed techniques and pixel design. A measured FWC of 103,448 electrons and MTF improvement of 300% are achieved with 6.5 μm pixel pitch.

## 1. Introduction

Imaging devices are essential components in the space environment for a range of applications including earth observation, star trackers on satellites, lander and rover cameras [1].

In space applications, charge coupled devices (CCDs) have been used for imaging devices because they can achieve low noise and high image quality. However, there are constraints for CCDs because of radiation tolerance, power, and size. The performance of CCDs is very sensitive to radiation in space. Charge transfer efficiency (CTE) of CCDs is degraded by proton irradiation, which causes position shifts of objects in images and leads star trackers to cause uncorrectable errors [2]. In addition, CCDs have a high power consumption because many external components and high supply voltages are necessary for their operation. These facts make CCDs less effective in the space environment. Therefore, CCDs have gradually been replaced by CMOS image sensors (CISs) due to their benefits, e.g., high level of integration, timing and control in a single chip, low power operation, and better radiation tolerance. CISs can produce high quality images by means of technical development, such as pinned photodiode (PPD), correlated double sampling (CDS), and recently developed circuit topology for low noise readout. Moreover, there is a growing interest in small spacecraft and satellites with lower weights and smaller sizes due to lower development costs and shorter lead times [3]. High integration ability of CISs is appropriate for small satellite missions. 

The main specifications of commercial CISs include quantum efficiency (QE), dynamic range (DR), saturation level, modulation transfer function (MTF), signal to noise ratio (SNR), dark current, image lag, non-uniformity, and non-linearity of the photon response. Among them, DR and MTF are especially important for space applications and these specifications should be maintained in radiation environments.

The DR is a quantity that describes the range of light intensity in an image. A high-DR image sensor can produce images with low to high light levels in an exposure time. It is defined as the ratio between the maximum saturated pixel output level and noise level in the dark [4]. The DR can be calculated as

(1) $$\mathrm{Dynamic}\;\mathrm{range}\;\left(\mathrm{DR}\right)=20\log\left(\frac{N_{\mathrm{sat}}}{N_{\mathrm{dark}}}\right)\left[\mathrm{dB}\right],$$

where N_sat_ is the number of electrons collected by a pixel at saturation level, which is also referred to as full well capacity (FWC), and N_dark_ is the number of electrons at noise level without illumination.

The improvement of DR is an important issue not only for image quality but also for accuracy of space sensor applications [5,6]. For example, star trackers use known star positions in an image taken with CISs on a spacecraft. An algorithm of the star tracker finds centroids of stars by using blurred spots. Because the wide dynamic range (WDR) of CIS makes the blurred spot more informative, the accuracy of the star tracker is increased with rapid determination of centroids [7].

Methods for achieving WDR can be categorized as a non-linear or linear. Previous reports have described DR extension with non-linear methods by several means. A well capacity adjusting method controlling an overflow gate caused low SNR because of the non-linear response of charge collection [8]. A logarithmic active pixel sensor using logarithmic response of MOS transistor in a weak inversion region caused high fixed pattern noise (FPN), which limits SNR because of large threshold voltage variation [9]. Linear-logarithmic pixel was also introduced to compensate the degradation of SNR, but the pixels were based on the three-transistor (3T) pixel structure, which has poor noise performance compared to a four-transistor (4T) pixel structure [10].

The linear method for the WDR is to increase FWC physically. In previous works [11,12], the FWC is required to be larger than 75,000 e^−^ for space applications. To satisfy this requirement, large pixel pitch was simply adopted. If high resolution is required, the chip size of CISs becomes large, so the manufacturing cost of the image sensor is increased and optical components also become bulky, which is not recommended for small satellite applications. Therefore, a demand for high FWC with small pixel pitch has been raised.

Modulation transfer function (MTF) is another important specification for quantifying image quality. It defines the ability of an optical system to resolve a contrast at spatial frequency. Higher MTF makes small objects distinguishable on images. For example, earth observation satellites, which observe geographical features, weather, and surveillance, require high MTF to distinguish over several meters or centimeters.

The major cause of MTF degradation is the crosstalk between neighboring pixels, especially for small-pitch pixels [13]. There can be both electrical and optical crosstalk depending on the cause. Electrical crosstalk is the phenomenon that photo-generated electrons move to the neighboring pixel through the substrate. This can be mitigated by shallow trench isolation (STI) and deep trench isolation (DTI) processes. Optical crosstalk is caused by undesirable photo generated electrons as a result of light incident in the lateral direction of the pixel. There are two approaches for degradation of optical crosstalk. The first approach prevents light from entering the PPD of adjacent pixels using a light guide, which refracts undesirable directions of light [14]. This significantly reduces the optical crosstalk but increases cost due to the additional process. Another way, which is adopted in this work, is to physically block the lateral light using a metal layer placed around edges of the PPD [15]. 

While most electrical parts equipped in a spacecraft have radiation shielding using aluminum or other metallic materials, CISs are directly exposed to outer space for image capturing. This requires CISs to have an extremely high level of radiation tolerance. The effects of radiation on CMOS technology, including MOSFET, capacitors, and various pixel structures, have been individually researched previously [16,17], but the pixel with an in-pixel capacitor has not been studied before. 

In this paper, a modified 4T pixel with a multiple charge transfer technique is proposed to extend the FWC without a non-linear response or SNR degradation. The measurement results show that the pixel can hold more than 100,000 electrons by means of the technique in a 6.5 μm pixel pitch. This value is approximately two times higher than in previously reported works [18,19,20,21,22,23]. Even though the MTF of a pixel can be improved either by using a specified fabrication process or design, these approaches could potentially increase the radiation damage effects. Therefore, metal shielding [24] was adopted to maintain the radiation tolerance of pixel devices. Two sensor chips were fabricated, one with metal shielded pixels and the other without metal shielding, and the measurement results show that an MTF improvement of approximately 307% was achieved by using metal shielding. In addition, the radiation damage effects of an in-pixel MOS capacitor are also investigated by comparing the performance of the sensor before and after irradiation.

The remainder of this paper is organized as follows. We describe the pixel configuration of the proposed CIS with an in-pixel MOS capacitor and its operation in Section 2. The prototype CIS implementation results, characteristics, and evaluation results are presented in Section 3. Finally, conclusions are given in Section 4.

## 2. Sensor Architecture

**Figure 1:**
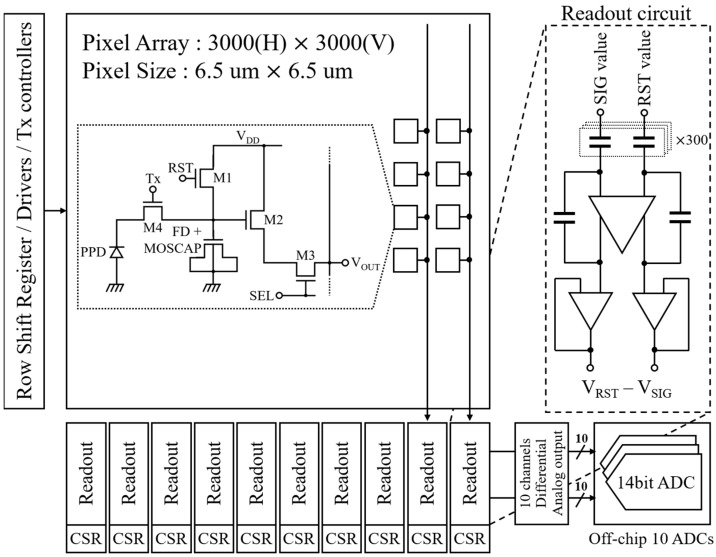
CMOS image sensor (CIS) block diagram. CSR, column shift register; ADCs, analog to digital converters.

Figure 1 shows a block diagram of the CIS and simplified readout circuit. The chip is comprised of a 3000 (H) × 3000 (V) pixel array, column readout circuits with buffer amplifiers, row shift registers, and Tx controllers.

There are 10 channel readout circuits in the CIS. The one channel of column readout circuits consists of a switched capacitor amplifier and unit gain buffer, which use a high gain operational amplifier. In one channel, 300 sets of two capacitors each were used for sampling reset and signal values out of the 300-pixel columns. A column shift register (CSR) sequentially selects one of 300 capacitor sets, and the sampled values are outputted in the form of analog voltage. The output voltages from the 10 channels are converted to digital values by ten off-chip analog to digital converters (ADCs). A row shift register (RSR) approaches each row in sequence starting at the first. The multiple charge transfer is controlled by the Tx controllers, which is similar to RSR. All pixel control signals are buffered by digital drivers.

### 2.1. Proposed Pixel Design with Multiple Charge Transfer

**Figure 2:**
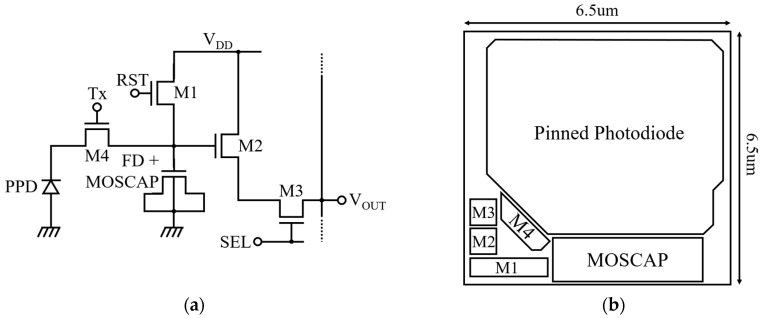
Proposed pixel for multiple charge transfer technique: (**a**) Schematic of the pixel; (**b**) Simplified pixel layout.

Figure 2a,b show a simplified schematic and layout of the proposed pixel. The pixel is similar to a conventional 4T pixel except that a single MOS transistor is added to the floating diffusion (FD) node as a charge storage capacitor for the multiple charge transfer technique. 

The pixel maintains beneficial properties of the conventional 4T pixel, such as low noise operation and high fill factor, unlike other specified pixel architectures for WDR operation [10,25]. Also, the pixel routing is simpler than in other WDR pixels because no additional control signal is required. In general, the FWC is limited by PPD capacitance, which depends on the PPD size and doping profile [26,27]. The PPD capacitance can be increased by expanding its size and adjusting the shape of the photodiode itself [4]. However, p-n junction capacitance of photodiode per unit area is not sufficient to increase the considerable amount of FWC within the limited pixel size. Increasing the doping profile of the PPD increases the PPD capacitance and FWC by reducing the depletion depth between the PPD and the substrate. However, only controlling the PPD doping cannot sufficiently increase the FWC for space applications.

In this work, instead of directly increasing the PPD capacitance, the FWC is enhanced by transferring photo-charges to the enlarged FD node multiple times. The use of an in-pixel MOS capacitor increases the capacitance of the FD node and photo-generated charge can be linearly integrated on the FD node without information loss.

**Figure 3:**
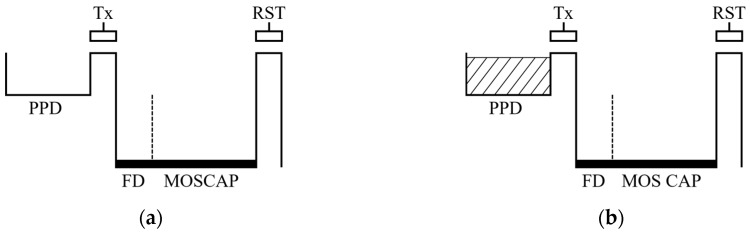

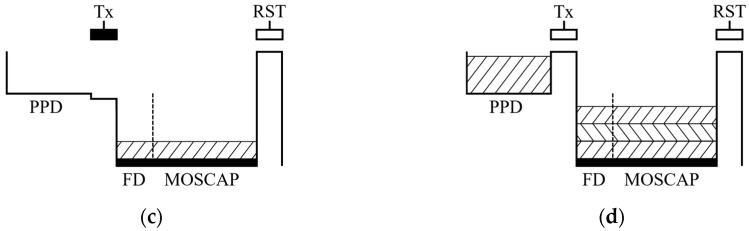
Potential diagram of the multiple charge transfer: (**a**) Initial potential of pinned photodiode (PPD) and floating diffusion (FD) node; (**b**) Integration of photo-generated charges in PPD; (**c**) Charge transfer to the FD node; (**d**) Accumulated charges in FD node after multiple charge transfer.

Figure 3 illustrates a potential diagram of the pixel for the multiple charge transfer. After the reset of the PPD and FD node as shown in Figure 3a, the integration of charges generated by photon is started. Before the integrated charges of the PPD are overflowed, the charges are transferred to the storage capacitor, as shown in Figure 3b,c. Figure 3d illustrates the output signal level after the charge transfer is repeated three times. The multiple charge transfer improves the SNR because signals generated from the photo charges tend to be correlated whereas noise is uncorrelated. For example, the signal and noise are increased by a factor of N and $\sqrt{N}$, respectively, where the N is the number of charge transfer. As a result, SNR is increased by a factor of $\sqrt{N}$.

### 2.2. Pixel Operation for Multiple Charge Transfer

**Figure 4:**
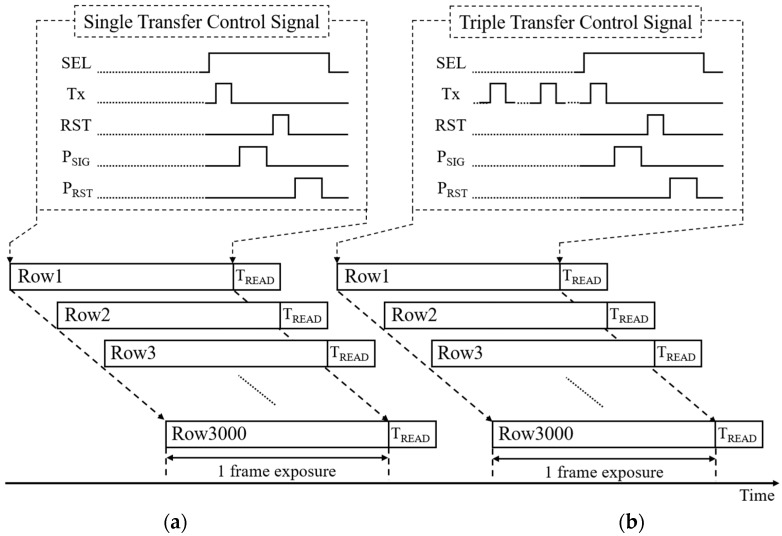
Timing diagram: (**a**) Single charge transfer; (**b**) Multiple charge transfer.

Figure 4a and b show the timing diagrams of single and triple charge transfer operations for the pixel control signals, respectively. The pixel readout process during T_READ_ will be explained in Section 2.4. First of all, one of the 3000 rows in the pixel array is selected by using the row select signal (SEL), and in case of single charge transfer, the photo-generated charge in the PPD is transferred to the FD capacitor when the transfer gate control signal (Tx) is high. The pixel signal value is then sampled by a sampling capacitor in the readout circuit when the signal sampling signal (P_SIG_) is high. After sampling the pixel signal value, the FD node is reset to an initial voltage when the RST is high. The reset value of the FD node is finally sampled using a sampling capacitor in the readout circuit for an analog delta reset sample (DRS) operation [28]. Starting from the first row, this procedure is repeated until the last row is processed.

The control signals for the multiple charge transfer operation are the same as the ones for the single charge transfer operation except that the Tx becomes high multiple times. As exemplified for triple charge transfer in Figure 4b, the photo-charge is transferred twice by making the Tx high two times before the SEL becomes high, and the last charge transfer is performed when the pixel row is selected, as with the single charge transfer operation.

Notice that the main difference from the conventional 4T pixel operation is that the operation order for the pixel reset and the photo-charge transfer is switched. While the photo-charge is transferred to the FD node after the reset in the conventional 4T pixel, the charge is transferred before the pixel reset, which allows for multiple charge transfer.

### 2.3. Pixel Design for Modulation Transfer Function

**Figure 5:**
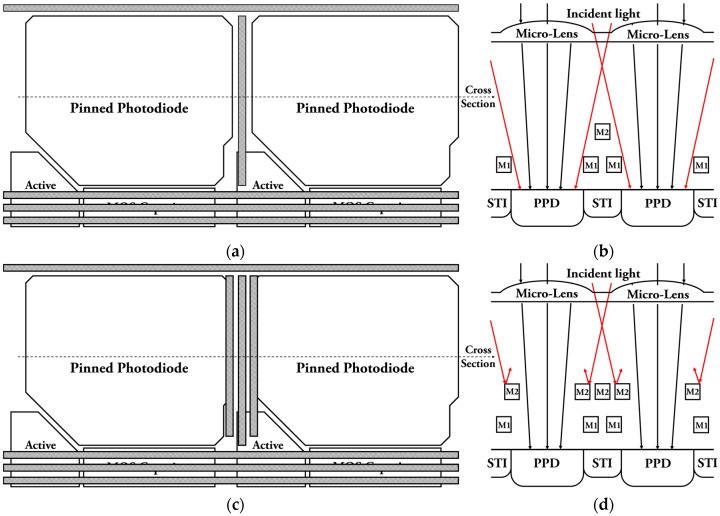
Layout of pixels and cross section view: (**a**) Proposed pixel layout including MOS capacitor without metal shielding; (**b**) Cross section view without metal shielding; (**c**) Proposed pixel layout with metal shield for enhancing MTF; (**d**) Cross section view with metal shielding.

The MTF, which can be measured in vertical and horizontal directions, is degraded by crosstalk between adjacent PPDs. One of effective ways to minimize crosstalk is to maximize the distance between the neighboring PPDs. However, this requires smaller PPD area for a limited pixel size, resulting in smaller fill factor and quantum efficiency (QE). Figure 5a,b show top-down and cross-section views of the two adjacent pixels. In Figure 5a, the PPD, active devices, and in-pixel capacitor are placed to maximize the PPD area. The control signals are routed horizontally not to interfere with the incident light for high QE. In this design, the vertical MTF is much better than the horizontal MTF because the distance between the adjacent PPDs in the vertical direction is much larger than that in the horizontal direction due to the active devices and in-pixel MOS capacitor placed under the PPD.

As shown in Figure 5b, the horizontal MTF is deteriorated by lateral light, and this cannot be improved by using STI. In order to improve the horizontal MTF without modifying the pixel design (especially the PPD size and shape, which were already optimized for optical and radiation performances), metal shielding [24] has been applied as shown in Figure 5c,d.

In this work, the layout of PPD, active devices, and MOS capacitor cannot be changed due to consideration of other parameters and radiation damage effects. Figure 5c shows a proposed method of metal routing for enhancing MTF performance. The cross-section view in Figure 5d shows the mechanism for blocking the lateral light by the shielding metals.

Since M1 was already used for the signal and power routings, as shown in Figure 5b,d, the space for shielding was insufficient. Even though the use of a stacked metal shielding (M2 and M3) helps to reduce the optical crosstalk, it also reduces the QE [29]. Therefore, only M2 was used for shielding in order to maintain the QE. The shielding metals can help to prevent generation of photo-charges near the PPD edges by blocking lateral light, so the horizontal MTF is improved. In addition, photo-charges that are generated in an active area can affect the pixel performance, so the active area is fully covered by metal layers to prevent the incident light. The measurement results show that the horizontal MTF has improved by three times from 0.131 to 0.402 with metal shielding.

### 2.4. Readout Circuit Design and Operation Procedure

**Figure 6:**
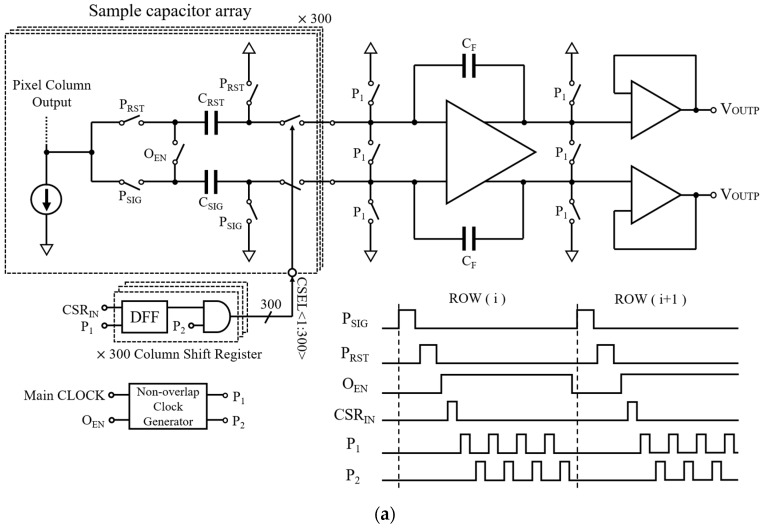

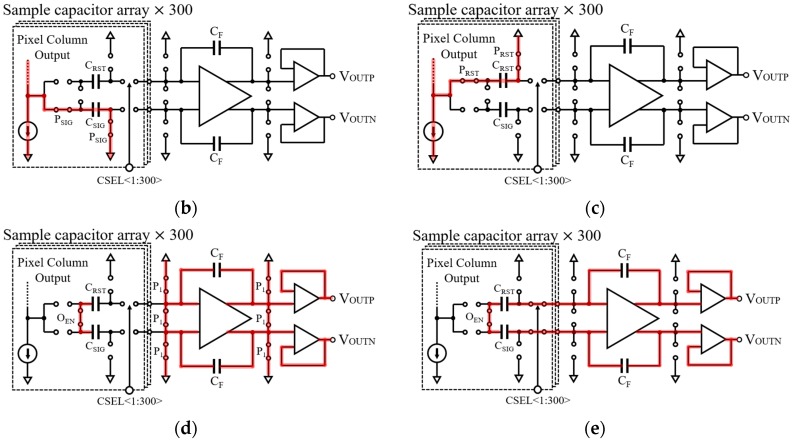
Readout circuit and timing diagram: (**a**) Overall readout circuit and timing diagram; (**b**) Signal sampling; (**c**) Reset value sampling; (**d**) Initializing the switched capacitor amplifier; (**e**) Differential output generation.

Figure 6a describes a readout circuit of a single channel and its control signals. One set of capacitors consisting of two sampling capacitors (C_SIG_ and C_RST_) is used to sample the pixel signal and reset values, respectively, from one-pixel column. Three hundred sets of sampling capacitors in one channel process the pixel values that are generated from 300 pixel columns. A switched-capacitor amplifier holds the sampled pixel values for the output buffer while also working as a variable gain amplifier (VGA). A two-phase non-overlapped clock generator generates the clock signals (P_1_ and P_2_) for the switched-capacitor amplifier. In order to sequentially transfer the sampled charges on the 300 sets of sampling capacitors, the clock signals are controlled by the column shift register (CSR), which is aligned with the CSR_IN_. 

The circuit operation is as follows. As explained in Section 2.2, the pixel signal and reset values are sampled on the C_SIG_ and C_RST_, respectively, while the P_SIG_ and P_RST_ are high. This is illustrated in Figure 6b,c. After sampling is completed, the output enable signal (O_EN_) becomes high and this shorts the bottom plates of the C_SIG_ and C_RST_, as shown in Figure 6d. Then, the C_SEL_ signal bus consisting of 300 control signals sequentially connects the top plates of the C_SIG_ and C_RST_ from the first set of sampling capacitors up to the 300th set to the input nodes of the amplifier. This makes the sampled charges on the C_SIG_ and C_RST_ move to the feedback capacitor (C_F_) and generate differential voltage outputs as follows.

(2) $$V_{\mathrm{OUTP}}=\frac{V_{\mathrm{RST}}-V_{\mathrm{SIG}}}{2},{\;V}_{\mathrm{OUTN}}=-\frac{V_{\mathrm{RST}}-V_{\mathrm{SIG}}}{2}.$$

Finally, the differential outputs are buffered by the two unit-gain amplifiers and routed to the off-chip ADC.

Notice that the proposed switched-capacitor amplifier performs a single-ended to differential signal conversion. The differential signaling is extremely important for space application because the off-chip ADCs are often placed far from the CIS in a shielded location due to the radiation damage effect. Therefore, the output signal traces become very long and are susceptible to common noises and interferences. The differential signaling helps to remove common noises and interferences effectively and maintain the signal integrity.

### 2.5. Radiation Damage Effects on the Proposed Pixel Structure

The effects of energetic particle radiation on CMOS devices and circuits in space can be categorized as total ionizing dose (TID) and displacement damage dose (DDD). The TID effect causes a build-up of trapped charge in the gate oxide of a CMOS device. The DDD effect occurs when high energy particles penetrate a silicon substrate. The energetic particles collide with silicon atoms and generate the dislocation of the silicon atoms which causes Frenkel pair defects. These effects cause a threshold voltage shift in CMOS devices and result in an excessive leakage current or a variation in device operating voltage.

In a CIS, the radiation damage effects deteriorate the optical performances, such as dark current, temporal noise, and random telegraph noise [30] or result in the generation of permanent hot pixel [31,32]. It is known that the radiation damage effect on CMOS devices is decreased as CMOS technologies shrink to deep sub-micron regimes and the oxide thickness becomes thinner. However, pixel devices do not benefit from advanced technologies because a thick gate oxide is still used in the pixel device.

In a conventional 4T pixel, the size of the transfer gate and PPD affects the dark current performance under radiation [33,34,35]. While small PPD size and short gate length help to reduce the radiation damage effect on the dark current, the FWC also decreases as a result of the reduced PPD capacitance. Moreover, the leakage current from the PPD to FD node can increase. In general, the radiation damage effect of a pixel device is reduced as the device size shrinks, and hence, the increment of dark current due to irradiation is alleviated [36]. Recent pixel design demands smaller pixel size in order to increase pixel resolution for a fixed chip area. Therefore, pixel design requires careful consideration of the trade-off between optical performance and radiation tolerance.

In this work, the pixel design followed previous researches for a conventional 4T pixel except for the in-pixel MOS capacitor. It has been reported that an amount of charges that can be trapped in an interface of SiO_2_/Si depend on the MOS capacitor size and radiation dose [37]. The trapped charges cause a shift in threshold voltage and variation of capacitance [17,37,38,39,40,41,42]. However, the radiation damage effect on a MOS capacitor used inside a pixel has not been studied yet. In order to analyze the effect of radiation on an in-pixel MOS capacitor, two different pixels having different sizes of capacitors has been fabricated and tested. The pixel performance under a radiation environment has been analyzed, and the analysis results are presented in the next section.

## 3. Experimental Results

### 3.1. Overall Performance with Enhanced Full Well Capacity

**Figure 7:**
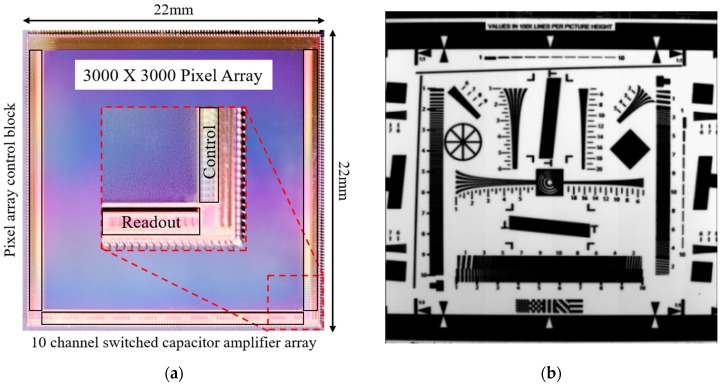
Chip microphotograph and captured image: (**a**) Chip microphotograph including a pixel array, pixel control block, and switched capacitor amplifiers of 10 channels; (**b**) Captured image (ISO 12233 chart).

The prototype CIS was fabricated using a 0.11 μm 1P4M CIS process. The chip microphotograph and the captured image of the ISO 12233 chart are shown in Figure 7a,b, respectively. 

**Figure 8:**
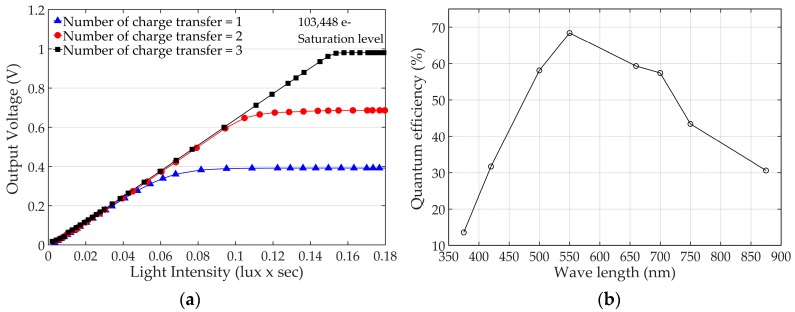
Measured pixel characteristic: (**a**) Readout voltages with a different amount of charge transfer; (**b**) Quantum efficiency chart with various wavelengths.

Figure 8a shows the measured pixel output voltages versus light intensity with different amounts of charge transfer. It is clearly shown that the saturation voltage of the pixel output linearly increases as the amount of charge transfer increases. The FWC of 103,448 e^−^ with the pixel readout voltage of 1 V was achieved by transferring the charges three times and the measured read noise—which was mainly generated by the off-chip ADCs and measurement board—was 24.7 e^−^. For the read noise measurement, the integration time of the CIS was set to 52 μs and the VGA gain to 4 for minimizing the dark and shot noise. Then, two dark images were captured, and the standard deviation was calculated from the difference of the two images for removing the fixed pattern noise. The read noise was calculated from the standard deviation considering ADC resolution, VGA gain, and conversion gain. The DR was achieved 72.4 dB resulting in the read noise. The DR could be further improved by using on-chip ADCs, which achieve much lower read noise. For example, a 106.7 dB DR can be achieved with 0.48 e^−^ read noise, which is reported in [43].

With a 6.5 um pixel pitch, the PPD size is 28.31 um^2^ and the fill factor is 67%. As shown in Figure 8b, the measured QE varies from 13.6% to 68.4% for the spectrum ranging from 375 nm to 875 nm, and the peak QE is 68.4% at 550 nm. Performance of the CIS is summarized in Table 1.

### 3.2. MTF Measurement Results

The MTF measurements have been made using the ISO 23333 slanted-edge method. The maximum output voltage of the bright pixel near the edge was limited to 50% of the saturated pixel output and the knife-edge was used as a target with a 5 degree tilting angle [24,44]. The MTF was measured in the vertical and horizontal directions and was expressed at Nyquist frequency. 

**Figure 9:**
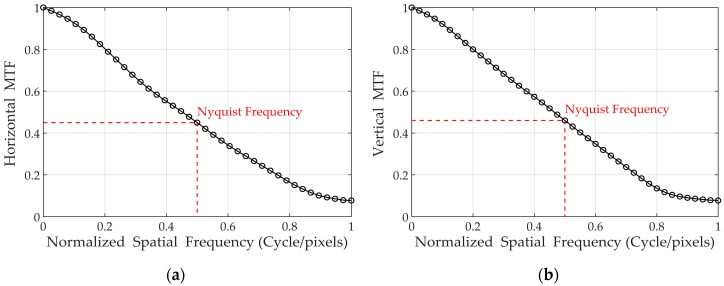
Measured MTF at 550nm wavelength: (**a**) Horizontal MTF; (**b**) Vertical MTF.

Two different CISs utilizing pixels with and without metal shielding have been fabricated in order to compare the MTF performance. Table 2 shows the MTF measurement results, and it clearly shows that the horizontal MTF has been increased from 0.131 to 0.402, resulting in a 300% improvement. The measured MTF curves with spatial frequency are also shown in Figure 9.

### 3.3. Radiation

**Figure 10:**
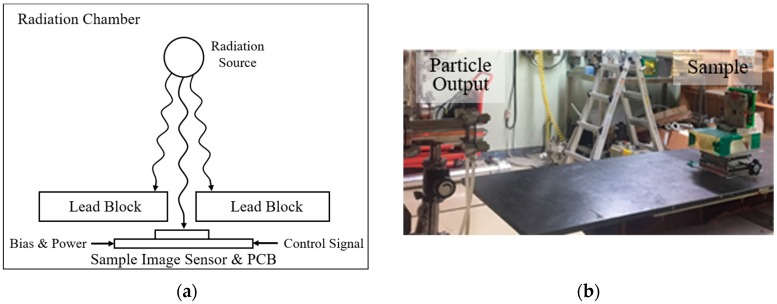

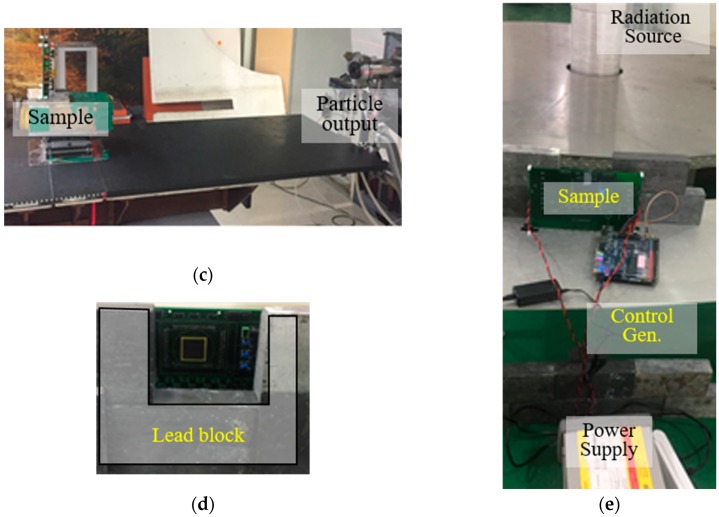
Radiation test setup: (**a**) Simplified block diagram; (**b**) Displacement damage dose (DDD) test with metal shielding to protect the other electrical parts; (**c**) DDD test setup without metal shielding; (**d**) Front view of total ionizing dose (TID) test setup using lead blocks; (**e**) Overall TID test setup including radiation source.

Figure 10 shows a simplified block diagram and actual pictures of the radiation test setup. The TID and DDD test setups are similar except for radiation source and exposure time. In the radiation chamber, the CIS chip is placed at a set distance from the radiation source, which is determined by the amount of radiation delivered to the chip per hour and electrical devices except for the CIS chip are protected from radiation by lead blocks. For the TID test, gamma rays generated by cobalt-60 (^60^Co) were used as a radiation source and the amount of radiation exposed to the chip was set to 236 rad per hour. Proton particles with a fluence of 1.48 × 10^10^ particles/cm^2^ are accelerated with an energy of 45 MeV by a MC-50 cyclotron, and the CIS chip was exposed to the accelerated proton particles for the DDD test.

**Figure 11:**
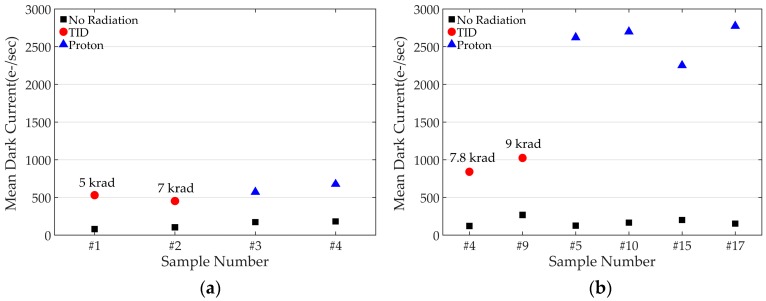
Radiation damage effects with two different in-pixel MOS capacitors: (**a**) 16.3 fF MOS capacitor (**b**) 19.6 fF MOS capacitor.

Three important performance measures (QE, power consumption, and dark current) are evaluated by the radiation test. While radiation did not affect QE and power consumption of the CIS, dark current was significantly increased depending on the size of in-pixel capacitor. The radiation damage effect on an in-pixel MOS capacitor on the dark current performance can be explained with the measurement results shown in Figure 11. The CIS with two different sizes of in-pixel MOS capacitors was fabricated and was tested for the TID and DDD. Figure 11a shows the test results for the CIS with an in-pixel MOS capacitance of 16.3 fF; the average dark current of CISs was 133.15 e^−^/s before the radiation test and it increased to 490.5 and 622.78 e^−^/s after the TID and DDD tests, respectively. With an in-pixel MOS capacitance of 19.6 fF, as shown in Figure 11b, the dark current significantly increased to 931.59 and 2585 e^−^/s for the TID and DDD test, respectively. Only a 20% increase in in-pixel MOS capacitance deteriorates the dark current performance by about two times for the TID and by four times for the DDD. The possible reasons for the sharp increase in dark current are the threshold voltage shift of the MOS capacitor due to the trapped charges in the oxide area and Shockley–Read–Hall (SRH) generation by the radiation induced defects. The measurement results indicate that there exists a critical trade-off between the FWC and the radiation damage effect on the dark current. With the multiple charge transfer technique, the FWC can be significantly enhanced by increasing the size of an in-pixel MOS capacitor, however, the dark current performance after radiation, especially for the DDD effect, can be severely worsened. Therefore, in-pixel capacitance should be carefully chosen based on an understanding of the trade-off.

Table 3 compares the radiation performance of this work with that of commercial image sensors which were tested with the similar radiation conditions. Dark current increment observed after irradiation of the commercial sensors varies from about 200% to 2700%, which is similar to the findings in this work.

## 4. Conclusions

A high FWC CIS for space applications was presented in this paper. The proposed pixel achieves high FWC by utilizing an in-pixel MOS capacitor and multiple charge transfer. In addition to the FWC, two important performance measures for space applications (MTF and radiation tolerance) were explained and a method to improve MTF was suggested. The radiation damage effect on an in-pixel MOS capacitor, which has not been reported in the other literatures, was studied by fabricating and measuring a CIS with two different size of the MOS capacitors. It was found that the size of the capacitor should be determined with consideration of the radiation damage effect because a small increment in the MOS capacitance can severely increase dark current under a radiation environment. The fabricated 3000 × 3000 CIS achieved an FWC of 103,448 electrons and metal shielding in the horizontal direction improved the MTF by 300%.

## Figures and Tables

**Table 1:** Characteristics of the CIS.

Parameter	Value
Technology	0.11 μm CMOS
Pixel Pitch	6.5 μm × 6.5 μm
Resolution	3000 × 3000 pixels
Chip size	22 mm × 22 mm
Fill factor	67%
Output voltage swing V_sat_ (gain=1)	1 V
Modulation transfer function (MTF) at Nyquist	0.4
Conversion gain	8.55 μV/e^−^
FD capacitance	19.6 fF
Full well capacity	103,448 e^−^
CIS read noise	24.7 e^−^
Dynamic range	72.4 dB
Quantum efficiency @ 550 nm	68.4%

**Table 2:** Measured MTF results with metal covering.

	MTF (Horizontal)	MTF (Vertical)
Metal covering	0.402	0.406
Without covering	0.131	0.406
Improvement (%)	307	0

**Table 3:** Comparison of the dark current with other image sensors (unit: e^−^/s).

	This work		[45]	[46]	[47]	[48]
Pre-Rad	133		6600	190	3135	20
TID * (Percentage increase)	491 (368%)	932 (700%)	180,000 (2727%)	1554 (818%)	6110 (195%)	N/A
Proton ** (Percentage increase)	623 (468%)	2585 (1941%)	N/A	N/A	N/A	500 (2500%)

* TID: total dose of 7~8 krad of cobalt-60 gamma ray. ** DDD: total proton fluence of 1 × 10^10^ ~ 1.5 × 10^10^/cm^2^ under 50 MeV.
